# Intraindividual development of MR lung perfusion parameters in children after congenital diaphragmatic hernia at 2 and 10 years

**DOI:** 10.1007/s00330-026-12443-5

**Published:** 2026-03-15

**Authors:** Greta Thater, Angelika Enns-Ercan, Julia Elrod, Schanas Jawhar, Michael Boettcher, Thomas Schaible, Christel Weiß, Stefan O. Schoenberg, Meike Weis

**Affiliations:** 1 Department of Radiology and Nuclear Medicine, University Medical Center Mannheim, Heidelberg University, Mannheim, Germany; 2 Clinic for Pediatric and Adolescent Surgery University Medical Center Mannheim, Heidelberg University, Mannheim, Germany; 3 Clinic for Neonatology and Pediatric Intensive Care Medicine, University Medical Center Mannheim, Heidelberg University, Mannheim, Germany; 4 Department of Medical statistics, Biomathematics and Information Processing, University Medical Center Mannheim, Heidelberg University, Mannheim, Germany

**Keywords:** Congenital diaphragmatic hernia, Lung hypoplasia, Lung perfusion, MR lung perfusion, Observed to expected lung volume

## Abstract

**Objective:**

This study aimed to analyze the intraindividual development of lung perfusion in children with congenital diaphragmatic hernia (CDH) at the ages of 2 and 10 years, as well as to investigate prenatal and postnatal influencing factors.

**Materials and methods:**

Fifty-nine children after CDH were examined as part of a monocentric follow-up program using dynamic contrast-enhanced MRI (DCE-MRI) at 2 years (hereafter referred to as Examination 1, E1) and again at 10 years of age (Examination 2, E2). Pulmonary blood flow (PBF) and pulmonary blood volume (PBV) were quantified separately for each lung. Additionally, prenatal parameters (observed-to-expected fetal lung volume, o/e FLV) and postnatal factors (extracorporeal membrane oxygenation (ECMO); fetoscopic tracheal occlusion (FETO); patch repair; recurrence; and reoperation for recurrence) were recorded.

**Results:**

Ipsilateral perfusion remained consistently reduced between E1 and E2 (63.4 ± 27.8 vs 62.0 ± 23.6 mL/100 mL/min; *p* = 0.8001), while PBV significantly decreased (*p* = 0.0213). Low prenatal o/e FLV values correlated with reduced ipsilateral PBF (E1: *r* = 0.51; *p* = 0.0082; E2: *r* = 0.03; *p* = 0.0342). Patients who underwent ECMO showed a decrease in contralateral PBF over time (*p* = 0.0435), and those with FETO tended to exhibit poorer perfusion courses.

**Conclusion:**

Patients with prenatally more severe lung hypoplasia, particularly those with low o/e FLV, exhibit persistently reduced lung perfusion even in the long term. These ongoing impairments remain stable over time, indicating permanently compromised lung development. Early identification and detailed risk assessment are therefore essential to initiate targeted therapeutic interventions.

**Key Points:**

***Question*** *Lung perfusion development in children with CDH between ages 2 and 10, including prenatal and postnatal influencing factors*.

***Findings*** *Ipsilateral lung perfusion remained reduced, PBV decreased, and low prenatal o/e FLV correlated with persistently impaired perfusion*.

***Clinical relevance*** *Children with severe prenatal lung hypoplasia show lasting perfusion deficits into adolescence. Early risk assessment enables timely, targeted interventions to mitigate long-term pulmonary impairment*.

**Graphical Abstract:**

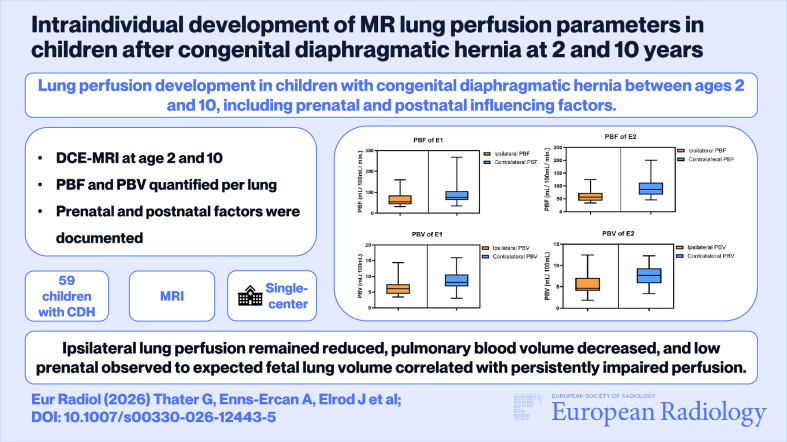

## Introduction

Congenital diaphragmatic hernia (CDH) occurs in 1–2 of every 3000 newborns, corresponding to an incidence of 0.03% [1].

Advances in prenatal and postnatal care have significantly improved survival rates, even in severe cases [2, 3]. However, increased survival is accompanied by long-term complications such as pulmonary dysfunction, developmental delays, feeding difficulties, and hernia recurrence [2]. These comorbidities burden healthcare providers and families, necessitating multidisciplinary management and structured follow-up [2, 4, 5]. Given the rarity and complexity of CDH, follow-up should be conducted in specialized centers to detect and manage long-term complications [6]. The University Medical Center Mannheim is one of Germany’s high-volume tertiary care centers, annually treating over 60 newborns with CDH and providing structured follow-up for 364 children.

Follow-up programs differ between centers, but the CDH Euro Consortium recommends systematic monitoring of pulmonary function, developmental delays, feeding, neurological outcomes, and recurrence risk [2, 3].

The consortium aims to establish standardized, multidisciplinary international follow-up programs [7].

Prenatal lung hypoplasia severity, estimated by observed-to-expected fetal lung volume (o/e FLV) on magnetic resonance imaging (MRI) or observed-to-expected lung-to-head ratio (o/e lung to head ratio) on ultrasound, remains the strongest predictor of postnatal morbidity and mortality [8–11].

Clinical interventions such as fetoscopic endoluminal tracheal occlusion (FETO) and extracorporeal membrane oxygenation (ECMO) provide further objective indicators of disease severity [12–14].

Assessment of postnatal lung development requires objective tools. Pulmonary function tests often require active cooperation, which can be challenging in young children. Imaging-based modalities offer compliance-independent alternatives. Among these, MRI provides advantages over scintigraphy: it is radiation-free and allows combined morphological and functional assessment [15, 16].

Dynamic contrast-enhanced MRI (DCE-MRI) enables quantification of perfusion parameters such as pulmonary blood flow (PBF) and pulmonary blood volume (PBV), allowing analysis of pathophysiological alterations in pulmonary perfusion [17, 18]. Previous studies by this research group demonstrated that children with CDH, including those requiring ECMO, showed reduced ipsilateral perfusion with lower PBF and PBV at ages 2 and 10 years, suggesting persistent impairment in pulmonary perfusion [18–21]. Scintigraphy studies similarly reported long-term perfusion asymmetry, often linked to functional limitations [22]. However, an intraindividual long-term analysis of individual perfusion development has not yet been performed, and its developmental course throughout childhood remains unclear.

Most available data are based on single age cohorts, limiting insight into the temporal development of pulmonary perfusion within CDH patients. Longitudinal analyses are therefore necessary to determine whether perfusion deficits remain stable, improve, or progress during childhood, and to evaluate how these changes relate to early predictors of severe pulmonary hypoplasia. Therefore, this study aims to analyze the intraindividual longitudinal development of pulmonary perfusion in a well-characterized CDH cohort, whose selected patients were taking part in a local follow-up protocol and were examined at the ages of 2 and 10 years, and to investigate associations with predictive markers of severe pulmonary hypoplasia [18–21].

## Material and methods

### Patient cohort

This retrospective single-center study included children with CDH who participated in the local follow-up program for children with CDH at the ages of 2 (Examination 1, E1) and 10 years (Examination 2, E2) between April 2008 and April 2022.

Of the 59 patients included in the present study, 54 had previously participated in studies conducted by this research group [18–21].

Inclusion criteria were complete participation in both examinations and sufficient MR image quality. Exclusion criteria were bilateral hernia, additional severe anomalies, incomplete follow-up, incomplete MRI protocols, motion artifacts, or inadequate documentation. Very preterm infants (< 34 weeks of gestation) were excluded, the cohort consisted predominantly of late preterm and term-born infants.

Of 59 patients included, 48 were eligible for perfusion analysis; in 11 patients, evaluation was not possible due to MRI limitation as incomplete MRI examination protocols and MRI images of insufficient evaluability due to motion artifacts.

Informed consent was obtained, and the study was approved by the local ethics committee (2024-415 M-§ 47 [3] MPDG).

### MRI-examination

The MRI acquisition protocols have been described in detail in previous publications from our research group. Briefly, examinations were performed on 1.5-T and 3.0-T scanners (MAGNETOM AERA and TRIO Siemens Healthineers) using a TWIST sequence (Time-resolved angiography With Interleaved Stochastic Trajectories); with view sharing (15% central, 20% peripheral k-space sampling); echo time 0.78 ms; repetition time 2.28 ms, flip angle 14, Generalized autocalibrating partially parallel acquisition factor 3, providing a 1.5 s temporal an 2 mm isotropic spatial resolution [21].

Lungs were segmented manually and separately on each side based on TWIST images, and macroscopic vessels in the hilar region were excluded from further analysis. This manual segmentation approach has previously shown good inter- and intra-observer agreement [20]. Perfusion values were reported as mean ± standard deviation for each lung side (ipsilateral referring to the side of the CDH, contralateral referring to the opposite side). Analysis was conducted using OsiriX software (OsiriX Versions 3.7.1, 3.8.1, and the FDA-approved version OsiriXPro 2.0—aycan Medical Systems, LLC).

Pulmonary perfusion was measured pixel-wise using a deconvolution method provided by a certified in-house perfusion plugin.

PBF (mL/100 mL/min) and PBV (mL/100 mL) maps were generated with the arterial input function obtained from a region of interest in the main pulmonary artery.

For E1, sedation with propofol was required, while E2 examinations were performed without sedation. 0.05 mmol/kg gadoteric acid (Dotarem, Guerbet) was used as a contrast agent, followed by a 10 mL saline (0.9% NaCl). Examination time was approximately 30 min.

### Clinical parameters

Recorded parameters included sex, age, hernia location, ECMO therapy, use of the FETO procedure, patch repair, hernia recurrence, reoperation, and the prenatal o/e FLV.

### Statistical analysis

Data was analyzed with GraphPad Prism (GraphPad Software, Insight Partners, Graphpad Holdings) and SAS software (Version 9.04, SAS Institute Inc.). Due to the non-normal distribution, Wilcoxon signed-rank tests were used for paired samples, and the Mann–Whitney *U*-test for independent samples. Correlations were assessed with Spearman coefficients; regression lines in scatter plots served as visual indicators only. Significance was set at *p* < 0.05.

## Results

### Descriptive data

Of the 59 patients, 25 were female and 34 male. 49 (83.05%) had left-sided hernias and 10 (16.95%) right-sided hernias. Mean o/e FLV was 27.4 ± 11.79%. Prenatally, 10.16% underwent FETO; postnatally, 58.18% required ECMO therapy. Patch repair was performed in 88.14%. Recurrence was diagnosed in 23.73%. For detailed information, see Table 1. Mean age at MRI was 24.75 ± 4.7 months (E1) and 124.60 ± 5.23 months (E2).

**Table 1:** Descriptive data

		Absolute number	Percent
Gender	Female	25	42.37
	Male	34	57.63
Localization of diaphragmatic hernia	Left	49	83.05
	Right	10	16.95
ECMO	Yes	32	58.18
	No	23	41.82
FETO	Yes	6	10.17
	No	53	89.83
Patch	Yes	52	88.14
	No	7	11.86
Recurrence	Yes	14	23.73
	No	45	76.27
Recurrence-operation	Yes	11	18.64
	No	3	5.08

Descriptive data (gender, hernia location, ECMO, FETO, patch-plastic repair, diaphragmatic hernia recurrence, and recurrence surgery)

*ECMO* Extracorporeal membrane oxygenation, *FETO* Fetoscopic tracheal occlusion

### Prenatal lung volume

**Fig. 1:**
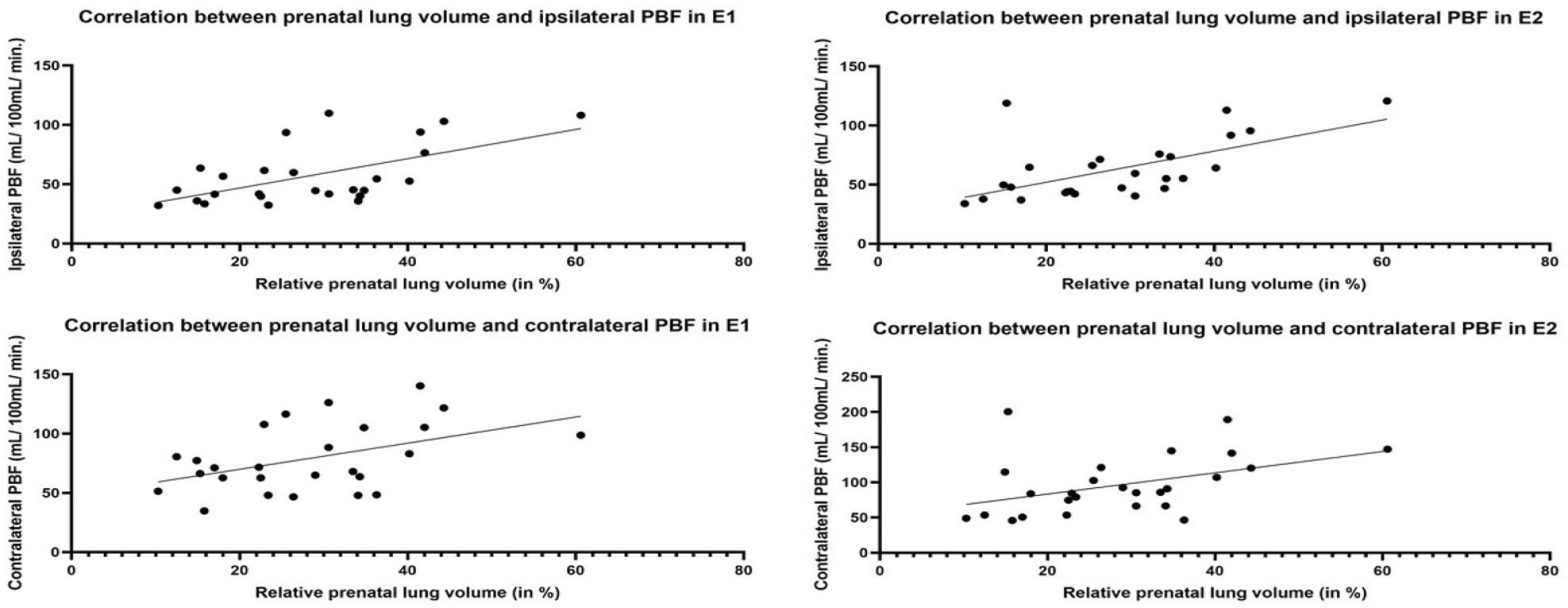
Correlation between prenatal lung volume and PBF (ipsilateral and contralateral) at the E1 and E2. PBF, Pulmonary blood flow

Higher o/e FLV correlated with higher postnatal PBF ipsilaterally (correlation coefficient *r* = 0.5073, *p* = 0.0082) and contralaterally (*r* = 0.3959, *p* = 0.0452) at E1, and ipsilaterally at E2 (ipsilateral: *r* = 0.342, *p* = 0.0342; contralateral: *r* = 0.1631, *p* = 0.5045), Fig. 1.

### Postnatal lung volume

**Fig. 2:**
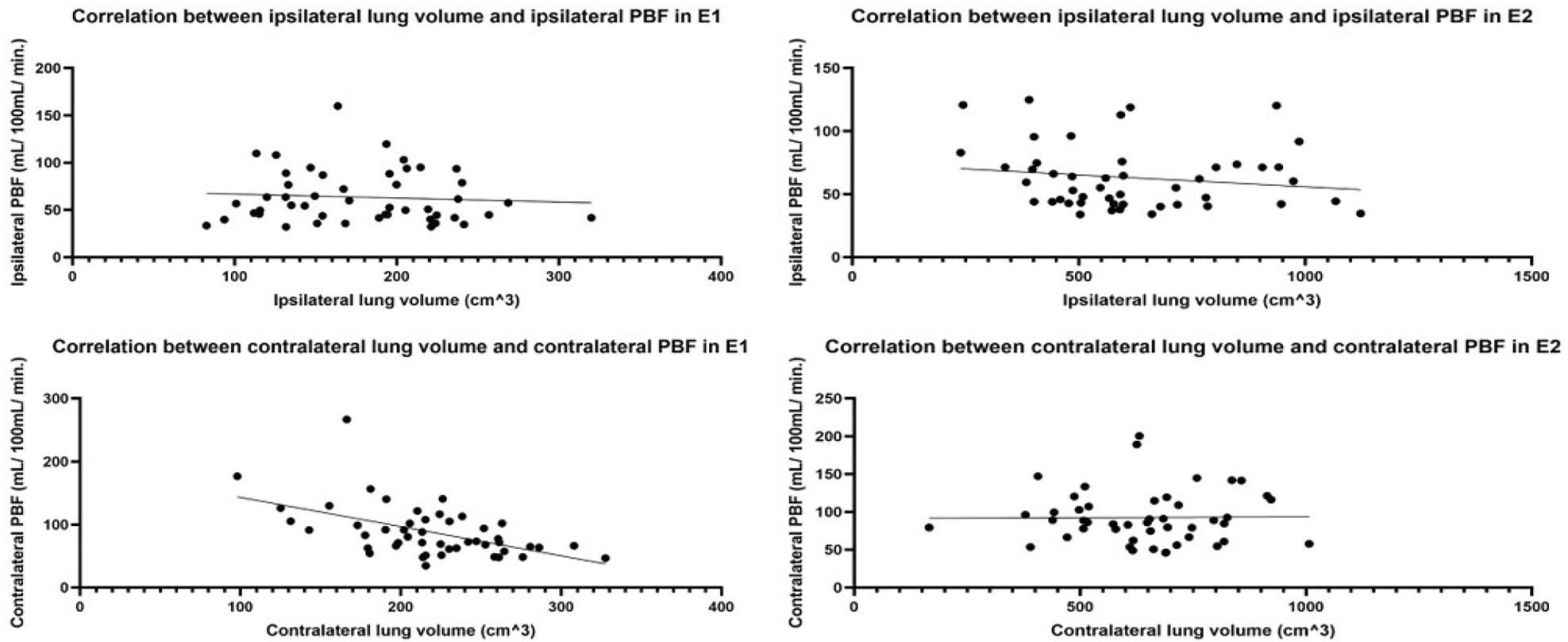
Correlation between lung volume and PBF (ipsilateral and contralateral) at the E1 and E2. PBF, Pulmonary blood flow

Correlation analysis demonstrated an inverse relationship between postnatal lung volume and PBF at both time points (Fig. 2).

At E1, a moderate statistically significant negative correlation was found contralaterally (*r* = −0.5047, *p* = 0.0003), whereas the ipsilateral correlation was weak and non-significant (*r* = −0.0843, *p* = 0.5687). Accordingly, higher lung volumes were associated with lower PBF, particularly on the contralateral side at E1.

At E2, the pattern shifted, a significant negative correlation was observed ipsilaterally (*r* = −0.4123; *p* = 0.0112), while the contralateral correlation was weaker and statistically non-significant (*r* = −0.2203; *p* = 0.1902). These findings suggest that negative correlations are present at both time points, with the side showing statistical significance differing between E1 and E2. Clinical factors, including ECMO (*p* = 0.5813) and FETO (*p* = 0.5548), showed no significant association.

Patch repair and hernia recurrence showed a non-significant trend towards reduced ipsilateral lung volume (patch repair *p* = 0.2954; presence of a hernia recurrence *p* = 0.3129). Additionally, contralateral lung volume was not associated with clinical outcome (recurrence *p* = 0.2277).

Patch repair showed no significant trend toward reduced ipsilateral lung volume (*p* = 0.0897) at E2. The influence on contralateral volume remained minor (*p* = 0.4198). The effects of ECMO therapy remained largely unchanged from E1 (ipsilateral *p* = 0.5707; contralateral *p* = 0.6198).

### Pulmonary perfusion parameters at the E1

**Fig. 3:**
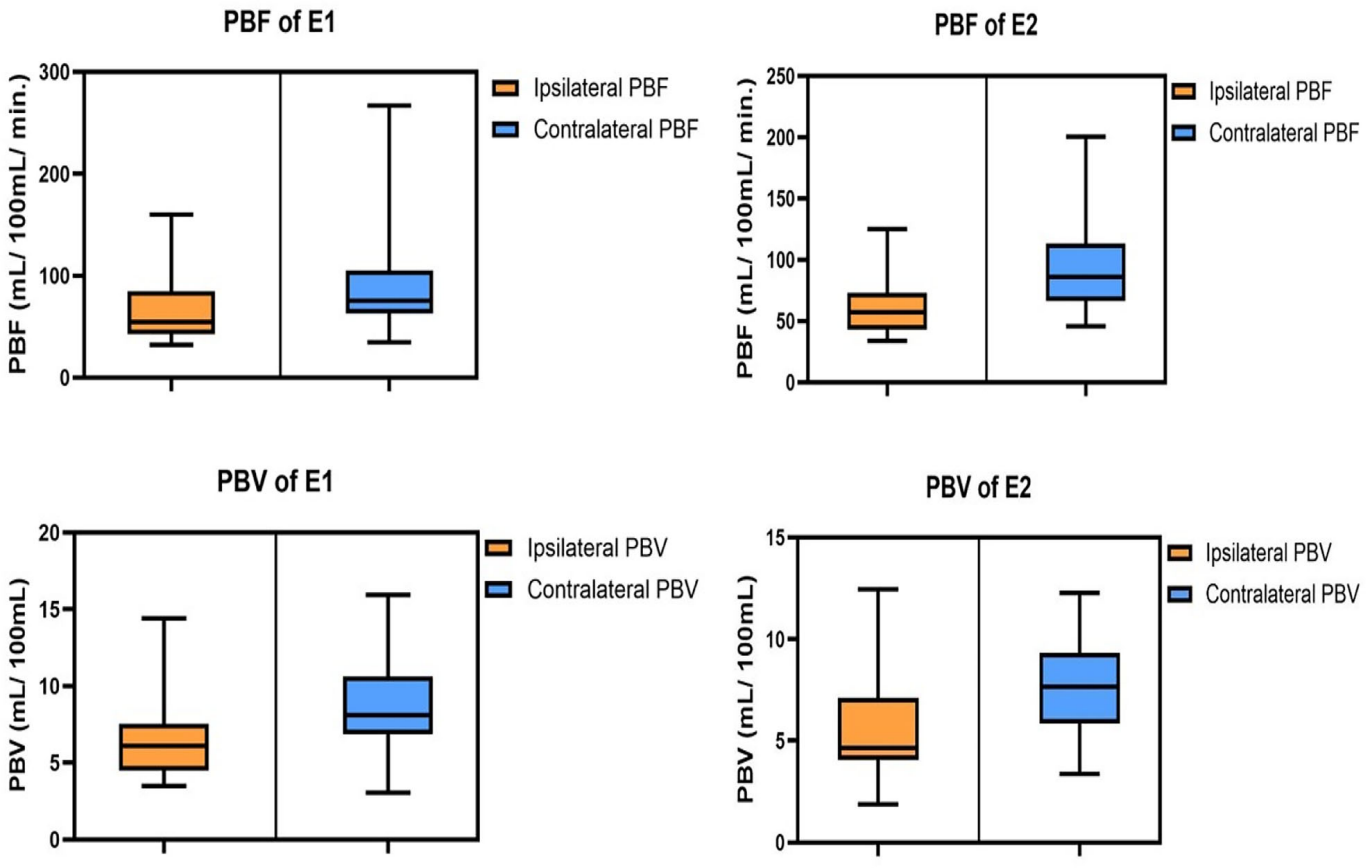
Comparison of PBF and PBV (ipsilateral vs contralateral) at the E1 and E2. The boxplots illustrate the PBF and PBV for the ipsilateral and contralateral lungs at the E1 (left) and E2 (right). The whiskers represent the 95% confidence interval. PBF, Pulmonary blood flow; PBV, Pulmonary blood volume

At E1, PBF was significantly lower on the ipsilateral side compared to the contralateral side (6.53 ± 27.77 mL/100 mL/min vs 88.32 ± 41.06 mL/100 mL/min, *p* < 0.0001), Fig. 3. Similarly, PBV was significantly reduced on the ipsilateral side (6.53 ± 2.47 mL/100 mL) compared to the contralateral side (8.78 ± 2.73 mL/100 mL, *p* < 0.0001), Fig. 3.

In children with postnatal ECMO therapy, mean ipsilateral PBF was tendentially lower (58.31 ± 26.99 mL/100 mL/min) compared to children without ECMO therapy (71.89 ± 27.16 mL/100 mL/min), though not statistically significant (*p* = 0.0611).

Prenatal FETO therapy showed no significant PBF difference between ipsilateral and contralateral lungs (61.36 ± 14.34 mL/100 mL/min ipsilateral, *p* = 0.7485 vs 96.36 ± 35.98 mL/100 mL/min contralateral, *p* = 0.3988), nor compared to patients without FETO (63.68 ± 29.04 mL/100 mL/min ipsilateral, *p* = 0.7485 vs 87.39 ± 41.89 mL/100 mL/min contralateral, *p* = 0.3988).

In patients with patch repair, the mean ipsilateral PBF was tendentially lower (61.18 ± 27.49 mL/100 mL/min) than with primary suture repair (82.86 ± 24.59 mL/100 mL/min), without statistical significance (*p* = 0.0544). Contralaterally, no PBF difference was observed between patch and primary suture repair (61.18 ± 27.49 mL/100 mL/min vs 82.86 ± 24.59 mL/100 mL/min).

Patients with and without hernia recurrence, as well as those with and without reoperation for recurrence, showed no significant differences in PBF, Table 2.

**Table 2:** Pulmonary perfusion parameters and clinical endpoints at E1 and E2

		Mean value		Standard deviation		*p*-value	
		PBF ipsilateral (mL/100 mL/min)	PBF contralateral (mL/100 mL/min)	PBF ipsilateral (mL/100 mL/min)	PBF contralateral (mL/100 mL/min)	PBF ipsilateral	PBF contralateral
E1							
Localization of CDH	Left	64.25	86.55	28.65	41.89	0.6289	0.2424
	Right	57.75	100.70	21.79	35.29		
ECMO	Yes	58.31	91.16	26.99	47.96	0.0611	0.9908
	No	71.89	85.75	27.16	29.48		
FETO	Yes	61.36	96.36	14.34	35.98	0.7485	0.3988
	No	63.68	87.39	29.04	41.89		
Patch	Yes	61.18	89.12	27.49	42.16	0.0544	0.7357
	No	82.86	81.43	24.59	32.91		
Recurrence	Yes	59.04	76.26	17.45	24.84	0.963	0.2859
	No	65.07	92.80	30.80	45.13		
Recurrence operation	Yes	55.23	71.90	15.61	17.72	0.5679	0.2089
	No	65.60	92.64	29.96	44.42		
E2							
Localization of CDH	Left	61.72	89.43	22.10	28.28	0.6776	0.6776
	Right	64.73	98.73	37.89	69.03		
ECMO	Yes	55.31	84.70	20.6	34.73	0.0365^a^	0.1198
	No	72.77	98.73	26.44	33.76		
FETO	Yes	47.41	69.84	11.19	25.12	0.1491	0.2123
	No	63.82	92.93	24.13	33.72		
Patch	Yes	58.67	88.79	19.17	31.67	0.0623	0.4526
	No	100.31	109.07	39.13	54.61		
Recurrence	Yes	52.64	75.75	14.71	22.22	0.1368	0.166
	No	65.53	95.88	25.43	35.51		
Recurrence operation	Yes	49.55	75.53	13.50	25.23	0.0916	0.2691
	No	64.96	93.91	24.59	34.47		

The table presents pulmonary perfusion parameters at the time of the E1 and E2 as mean ± standard deviation, along with the corresponding *p*-values for the ipsilateral and contralateral lungs in relation to various clinical endpoints

^a^ Statistically significant

*CDH* congenital diaphragmatic hernia, *ECMO* Extracorporeal membrane oxygenation, *FETO* Fetoscopic tracheal occlusion, *PBF* Pulmonary blood flow

### Pulmonary perfusion parameters at the E2

At E2, mean ipsilateral PBF was significantly lower than contralateral (62.04 ± 23.55 mL/100 mL/min vs 90.44 ± 33.41 mL/100 mL/min, *p* < 0.0001). PBV also remained significantly reduced on the ipsilateral side compared to the contralateral side (5.54 ± 2.29 mL/100 mL vs 7.66 ± 2.32 mL/100 mL, *p* < 0.0001), Fig. 3.

**Fig. 4:**
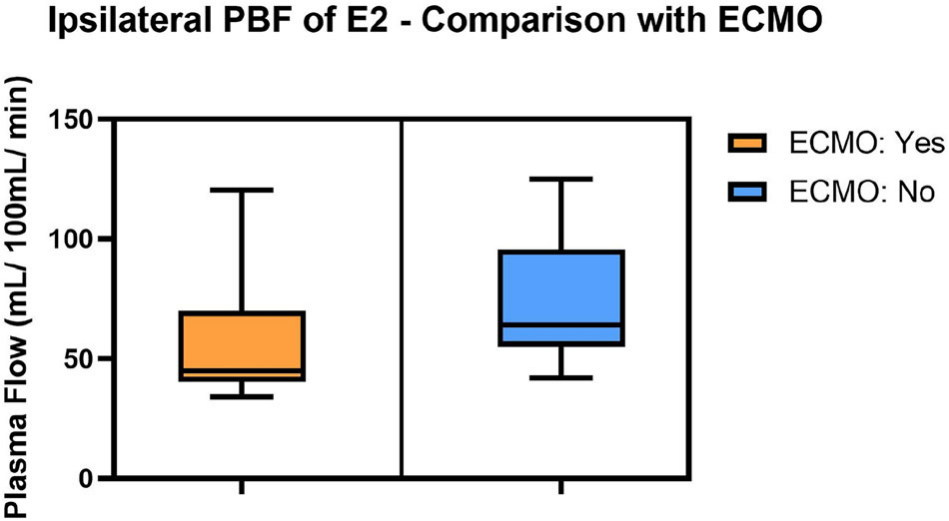
PBF ipsilateral and ECMO at the E2. The boxplots display PBF under the influence of ECMO at the E2 examination. The whiskers represent the 95% confidence interval. ECMO, Extracorporeal membrane oxygenation; PBF, Pulmonary blood flow

In children with postnatal ECMO, ipsilateral PBF was significantly lower than without ECMO (55.31 ± 20.6 mL/100 mL/min vs 72.77 ± 26.44 mL/100 mL/min, *p* = 0.0365), Fig. 4. Contralateral PBF tended to be lower after ECMO than without ECMO (84.7 ± 34.73 mL/100 mL/min vs 98.73 ± 33.76 mL/100 mL/min, *p* = 0.1198).

No significant perfusion differences were found between children with and without prenatal FETO (Table 2).

In patch repair, ipsilateral PBF tended to be lower than with other repair types (58.67 ± 88.79 mL/100 mL/min vs 100.31 ± 39.13 mL/100 mL/min, *p* = 0.0623). Patients with hernia recurrence showed no significant differences in PBF, either ipsilateral or contralateral, nor did those require reoperation (Table 2). Hernia side (left vs right) had no relevant impact on lung perfusion (Table 2).

### Intraindividual development of pulmonary perfusion parameters from E1 to E2 and the association of clinical parameters with the change in pulmonary perfusion parameters

Longitudinal analysis of pulmonary perfusion parameters from E1 to E2 revealed stable PBF ipsilateral (E1: 63.44 ± 27.77 mL/100 mL/min; E2: 62.04 ± 23.55 mL/100 mL/min, *p* = 0.8001) and contralateral (E1: 88.32 ± 41.06 mL/100 mL/min; E2: 90.44 ± 33.41 mL/100 mL/min, p = 0.7071).

PBV decreased significantly ipsilaterally (E1: 6.53 ± 2.47 mL/100 mL; E2: 5.54 ± 2.29 mL/100 mL, *p* = 0.0213), while contralateral values showed a nonsignificant decrease (E1: 8.78 ± 2.73 mL/100 mL; E2: 7.66 ± 2.32 mL/100 mL, *p* = 0.2263).

Hernia localization had no significant impact on PBF development (ipsilateral: *p* = 0.5123, contralateral: *p* = 0.1332). After prenatal FETO therapy, contralateral lung perfusion tended to decline compared to children without FETO (–24.92 ± 37.72 mL/100 mL/min vs 7.763 ± 48.99 mL/100 mL/min, *p* = 0.079, Table 3). A similar but nonsignificant trend was observed on the ipsilateral side (*p* = 0.0921).

Following ECMO, contralateral PBF decreased significantly (ECMO yes: −6.177 ± 55.1 mL/100 mL/min, ECMO no: 17.14 ± 34.8 mL/100 mL/min, *p* = 0.0435), while ipsilateral PBF remained unchanged (ECMO yes: −1.684 ± 27.28 mL/100 mL/min, ECMO no: 0.08947 ± 22.87 mL/100 mL/min, *p* = 0.7588), Table 3.

Other postnatal factors, such as hernia recurrence and reoperation, were associated with trends toward reduced lung perfusion without statistical significance (Table 3). For example, children with hernia recurrence showed ipsilateral decline of PBF (−6.84 ± 15.73 mL/100 mL/min; *p* = 0.1101) and a slight contralateral decrease (recurrence yes: −1.196 ± 27.53 mL/100 mL/min, recurrence no: 6.423 ± 54.67 mL/100 mL/min; *p* = 0.3579). Similar non-significant findings were seen in reoperated patients (reoperation yes: 2.82 ± 25.21 mL/100 mL/min, reoperation no: 4.77 ± 53.40 mL/100 mL/min; *p* = 0.6617).

**Table 3:** Pulmonary perfusion development from E1 to E2

PBF E2–E1		Mean value		Standard deviation		*p*-value	
		ipsilateral	contralateral	ipsilateral	contralateral	ipsilateral	contralateral
Localization of CDH	Left	−0.09663	6.078	24.34	44.93	0.5123	0.1332
	Right	−2.163	−7.674	33.28	74.15		
ECMO	Yes	−1.684	−6.177	27.28	55.10	0.7588	0.0435
	No	0.08947	17.14	22.87	34.80		
FETO	Yes	−16.6	−24.92	22.45	37.72	0.0921	0.079
	No	1.534	7.763	25.06	48.99		
Patch	Yes	−1.642	1.106	25.87	49.39	0.3555	0.079
	No	10.71	32.34	16.37	33.35		
Recurrence	Yes	−6.842	−1.196	15.73	27.53	0.1101	0.3579
	No	2.054	6.423	27.73	54.67		
Recurrence-operation	Yes	−5.332	2.82	15.89	25.21	0.2741	0.6617
	No	0.9547	4.765	27.15	53.4		

The table presents the development of pulmonary perfusion from E1 to E2 as mean ± standard deviation, along with the corresponding *p*-values for the ipsilateral and contralateral lungs across the various endpoints

*CDH* congenital diaphragmatic hernia, *ECMO* Extracorporeal membrane oxygenation, *FETO* Fetoscopic tracheal occlusion, *PBF* Pulmonary blood flow

## Discussion

This study demonstrates persistent differences in lung perfusion between the ipsilateral and contralateral lungs in children after CDH. Across the cohort, ipsilateral PBF remained significantly reduced compared with the contralateral side from 2 to 10 years, while intraindividual perfusion values were largely stable from early childhood to late follow-up. These findings suggest lasting asymmetry in lung perfusion, longitudinal changes within individuals were generally small.

ECMO therapy and patch repair were associated with lower lung perfusion values over time, particularly ipsilateral. These associations, especially the significantly reduced ipsilateral PBF after ECMO, should not be interpreted as a causal effect of the interventions themselves, but rather as a marker of high initial disease burden. Thus, the functional impairments reflect underlying disease severity [12, 23]. This interpretation is consistent with previous studies like Spoel et al, who demonstrated using spirometry that CDH infants, particularly post ECMO therapy, had reduced lung function, increased lung volumes, growth retardation, and respiratory diseases [24]. Similar results were reported by Weis et al and Toussaint-Duyster et al, who attributed long-term impairments to pronounced pulmonary hypoplasia with limited growth potential, vascular malformations, and pulmonary hypertension [12, 25].

While contralateral PBF declined significantly from E1 to E2 after ECMO, the magnitude of change was moderate ipsilaterally and showed considerable variability. This finding may reflect increased compensatory strain of the contralateral lung and/or persistent and potentially increasing pulmonary hypertension in the context of severe pulmonary hypoplasia; however, given the heterogeneity and limited sample size, this interpretation remains speculative.

Prenatal FETO therapy showed no significant long-term impact on PBF. The tendency towards reduced bilateral PBF at E2 likely reflects greater baseline disease severity rather than an adverse effect of the intervention itself, particularly considering the small number of patients treated with FETO in this cohort. Therefore, conclusions regarding long-term perfusion effects of FETO should be drawn with caution.

Patch repair was associated with a tendency toward reduced ipsilateral PBF at both time points, consistent with Panitch et al, who described impaired lung function in children with patch repair up to the age of three [26]. Since patch repair is used for larger defects, reduced PBF mainly reflects the severity of pulmonary hypoplasia rather than an independent effect on lung perfusion [27]. Hernia recurrence and reoperation were associated with trends toward reduced lung perfusion both ipsilaterally and contralaterally. The absence of measurable improvement after reoperation suggests that pronounced pulmonary hypoplasia in large diaphragmatic defects may limit functional recovery even after anatomical correction [27].

Prenatal o/e FLV correlated significantly with postnatal pulmonary perfusion, particularly on the ipsilateral side, and remains a stable prognostic marker over time. Higher prenatal relative lung volume predicted better postnatal PBF, supporting the pathophysiological hypothesis that intrauterine lung development provides the structural basis of later lung function, which can only be modified to a limited extent by postnatal interventions up to now. At E2, the association persisted only on the ipsilateral lung, underscoring the limited but lasting prognostic value of prenatal lung volume rather than a uniform long-term prediction [28–30].

Postnatally measured lung volume demonstrated weak to moderate inverse correlation with PBF, with statistical significance varying by side and time point. Although counterintuitive, this inverse relationship may be explained by the fact that postnatal lung volume likely reflects aeration rather than perfusion and that relative hyperinflation may limit PBF [31].

Similar inverse associations have been reported by previous studies in patch repair patients, possibly due to mechanical restriction of thoracic growth [26]. In the present study, patch repair showed a non-significant trend toward reduced ipsilateral postnatal lung volume, while no relevant association with contralateral lung volume was observed [26].

However, no direct link between the patch use and thoracic or scoliotic deformities has been demonstrated in the literature so far. Koziara et al suggest that scoliosis in CDH patients usually develops before the age of 10, often part of a multisystemic disease, with congenital vertebral anomalies being rare [32]. Russell et al reported increased rates of scoliosis and chest wall deformities in children with severe CDH, independent of repair technique [33].

Postnatal ECMO therapy had only a moderate effect on lung volume but showed a marked impact on pulmonary perfusion.

Overall, MR-based pulmonary perfusion remained largely stable intraindividually from E1 to E2 with modest changes primarily affecting PBV. Hofhuis et al similarly reported stable lung volumes and persistently reduced yet stable spirometry function at 6 months and 1 year [34]. Hayward et al found a persistent ventilation/perfusion (V/Q) mismatch in 61% of children with patch repair linked to a higher risk [35]. Our results confirm that ECMO and patch reconstruction are associated with lower intraindividual lung perfusion, primarily as markers of disease severity. Several limitations of this study should be acknowledged. First, different MRI field strengths (1.5 T and 3 T) were used, which may affect perfusion quantification due to differences in signal-to-noise ratio, T1 relaxation, and flow sensitivity. Although no systematic differences were observed in our cohort, future studies could further minimize the impact of scanner-specific effects by calculating the ratio of ipsilateral to contralateral perfusion, implementing scanner-specific calibration, or performing multicenter analyses with field strength correction.

Second, manual segmentation was applied, which may introduce observer dependency despite previously demonstrated reproducibility. Future studies should focus on automated segmentation approaches to improve robustness [20].

Third, the observed PBV changes were modest in absolute terms and showed variability, suggesting that technical measurement variability may have contributed to the findings. Thus, the significant ipsilateral PBV decrease, corresponding to a relative change of 23%, likely reflects a genuine physiological effect, although this should be interpreted with some caution.

In summary, pulmonary perfusion asymmetry persists into late childhood of CDH patients with reduced ipsilateral perfusion, indicating lasting functional impairments. Prenatal lung hypoplasia, ECMO therapy, and hernia recurrence, are associated with lower pulmonary perfusion, primarily as markers of disease severity. While severe pulmonary hypoplasia detected prenatally (low o/e FLV) is a predictor of reduced pulmonary perfusion, clinical factors often show weak associations with perfusion. MRI offers a radiation-free method for longitudinal perfusion assessment; however, prospective multicenter studies are required before these findings can build evidence-based guidelines.

## Abbreviations

DCE-MRI: Dynamic contrast-enhanced MRI
E: Examination
ECMO: Extracorporeal membrane oxygenation
FETO: Fetoscopic tracheal occlusion
MRI: Magnetic resonance imaging
o/e FLV: Observed to expected fetal lung volume
PBF: Pulmonary blood flow (mL/100 mL/min)
PBV: Pulmonary blood volume (mL/100 mL)
TWIST: Time-resolved angiography with interleaved stochastic trajectories
